# Ultrasound for degradation of complex matrices of PFAS mixtures

**DOI:** 10.1007/s11356-025-37055-2

**Published:** 2025-10-15

**Authors:** Jay N. Meegoda, Bruno Bezerra de Souza, Targol Teymourian, Duwage C. Perera, Purshotam Juriasingani, Jeffrey Davis

**Affiliations:** 1 New Jersey Institute of Technology (NJIT), Newark, NJ USA; 2 Tetra Tech, Inc, Pasadena, CA USA; 3 Air Force Civil Engineering Center, San Antonio, TX USA

**Keywords:** Ultrasound technology, PFAS degradation, Still bottom, Groundwater, AFFF, Advanced oxidation

## Abstract

This study evaluates the effectiveness of ultrasound technology for degrading per- and polyfluoroalkyl substances (PFAS) in three complex environmental matrices: groundwater (GW), still bottom (SB), and aqueous film-forming foam (AFFF). A 10-L ultrasonic reactor, equipped with multi-frequency piezoelectric elements (850 kHz and 950 kHz), was used to treat PFAS-contaminated samples for 6 to 12 h. Degradation efficiency was measured using liquid chromatography-mass spectrometry (LC–MS/MS), fluoride ion-selective electrode (F-ISE), suppressed conductivity ion chromatography (IC), nuclear magnetic resonance (NMR) spectroscopy, total organically bound fluorine (TOF) analysis, and inductively coupled plasma mass spectrometry (ICP-MS). LC–MS/MS confirmed PFAS degradation, while F-ISE quantified fluoride release, indicating defluorination. IC analysis measured changes in anion concentrations, particularly sulfate and chloride, to assess transformation pathways. NMR and TOF provided structural insights into PFAS breakdown, and ICP-MS tracked variations in metal concentrations, highlighting potential interactions with degradation byproducts. In SB samples, fluoride concentration increased from 0 to 8.71 mg/L after 12 h, indicating successful defluorination of PFAS compounds. For GW samples, fluoride levels rose moderately from 0.54 to 1.78 mg/L, demonstrating that sonolysis can degrade PFAS in lower-concentration matrices. However, AFFF samples, dominated by perfluorooctanesulfonic acid (PFOS), showed only a slight increase in fluoride concentration (0.75 to 1.37 mg/L), indicating resistance to sonolytic degradation due to strong carbon–fluorine bonds. Anion and metal analysis revealed matrix-specific interactions influencing sonolysis outcomes, with energy distribution analysis highlighting the competitive role of chemical oxygen demand (COD) in scavenging reactive radicals. This research demonstrates ultrasound as a promising technology for PFAS degradation in complex matrices. However, the test results for AFFF suggest that with high surfactant concentrations, modifications may be necessary for complete mineralization of PFAS compounds.

## Introduction

Per- and polyfluoroalkyl substances (PFAS) have gained enormous industrial significance and are now widely found in various consumer goods due to their remarkable chemical and thermal stability (Li et al. 2023). A vast array of pervasive film-forming PFAS compounds and their derivatives have been continuously developed and released into the global market since the early 1940 s, impacting many facets of human existence (Loganathan et al. 2024; Malovanyy et al. 2023). At present, the market offers more than 6500 different types of synthetic perfluorinated chemicals (Sharma et al. 2024). Furthermore, because of their hydrophilic (polar head) and lipophilic (fluorinated tail) groups, PFAS exhibit unique partitioning behavior in the environment. Excellent chemical and thermal stability of PFAS is provided by their robust carbon–fluorine (C–F) bonds (544 kJ/mol) (Li et al. 2023; Sabba et al. 2024; Vakili et al. 2024). Therefore, they have a long half-life in the environment, and their hydrophobic and oleophobic properties as well as their chemical and mechanical stability, make them extremely resistant to oxidation and degradation (Taher et al. 2024; Venkatesh Reddy et al. 2024). Because of these well-known characteristics which have earned PFAS the moniker “forever chemicals,” they pose a serious environment threat due to their persistence, toxicity, and mobility (Buttle et al. 2023; Miglio et al. 2024; Perera & Meegoda 2024).

The detrimental effects of both legacy PFAS, like the well-researched perfluorooctanesulfonic acid (PFOS) and perfluorooctanoic acid (PFOA), as well as developing PFAS, also known as persistent organic pollutants, have been widely documented in the literature (Manojkumar et al. 2023). Concern has been further heightened by growing evidence that PFAS can enter the food chain and undergo biomagnification (Donley et al. 2024; Iannone et al. 2024; Niu et al. 2024; Vázquez Loureiro et al. 2024). This emphasizes the urgent need to close the research gap on PFAS detection and treatment (Liu et al. 2024; Zhang et al. 2024). PFAS exposure can occur through multiple routes, including skin contact, inhalation, and ingestion, allowing these compounds to accumulate in living organisms, including humans (Munoz et al. 2022).

Stationary and discrete structures, such as industrial facilities, landfills, water and wastewater treatment facilities, and fire training grounds, are point sources of PFAS (Goukeh et al. 2025; Roy et al. 2025; Thelusmond et al. 2025). On the other hand, nonpoint sources include air movement and the use of industrial and consumer goods (Cui et al. 2020). Because of their extensive use, high persistence, and capacity for bioaccumulation, pervasive formal and semi-solid PFAS pollutants have spread throughout a variety of environmental matrices. This has sparked worries about how they may affect human health, leading to problems like neurodevelopmental abnormalities, compromised immune systems, unfavorable pregnancy outcomes, and elevated risks of cancer (Lenka et al. 2021; Zhou et al. 2021, 2023).

The harmful effects of PFAS exposure have been known for decades, but efforts to address these risks have been delayed (Bline et al. 2024; Coperchini et al. 2024; Dias et al. 2024; Niu et al. 2024). Industry-led studies on PFAS treatment methods, combined with socioeconomic factors, have hindered progress in removing PFAS from the environment. According to Gaber et al. (2023), the main challenge in achieving effective PFAS remediation is not a lack of viable solutions, but the absence of transparency of industry-sponsored research. Their analysis of previously secret industry documents shows that the dangers of PFAS were identified and deliberately hidden long before being published in scientific journals (Gaber et al. 2023).

PFAS can be removed from drinking water using separation technologies like granular activated carbon (Abulikemu et al., n.d.; Nakazawa et al. 2024; Ranjbar et al. 2024) and ion exchange resin (Liang et al. 2022; Liu et al. 2022). Large volumes of water with low PFAS concentrations typically in the parts per trillion (ppt) range are fed into separation technologies, which separate the influent into two streams: one with high PFAS concentrations (often referred to as the brine solution, still bottoms (SB), or high concentration PFAS waste) and another with purified water. Along with salts and other organic compounds, this waste stream may contain PFAS in the parts per million (ppm) range (Marsh et al. 2024; Singh et al. 2020; Wang et al. 2021).

To make sure the extremely concentrated PFAS waste does not end up back in the environment, it needs to be processed further, in order to eliminate the health hazards to the public (Kewalramani et al. 2022; Marsh et al. 2024). Concentrated PFAS could be destroyed by incineration; however, in 2020, the United States Environmental Protection Agency (USEPA) released a technical brief on the topic, the main finding of which was that it is unclear how well PFAS can be destroyed by incineration and what happens to them in terms of possible mixed fluorinated organic byproduct formation (Chiang et al. 2023; Krause et al. 2021; Singh et al. 2021; Chowdhury et al. 2021; Verma et al. 2024; Smith et al. 2023; Gomez-Ruiz et al. 2017; Kewalramani et al. 2022).

Unlike conventional high-temperature incineration, which operates in the gas phase at sustained temperatures above 1000 °C and poses risks of incomplete combustion and toxic emissions, sonolysis leverages localized, short-lived high-temperature zones (~ 5000 K) generated during acoustic cavitation in liquid media. These implosive microbubble collapses occur at ambient bulk temperature, enabling PFAS mineralization without the need for external bulk heating or the formation of hazardous byproducts. Thus, while both techniques rely on heat for PFAS destruction, their mechanisms, media, and operational conditions differ significantly (Campbell & Hoffmann 2015; Kewalramani et al. 2022, 2023; Kewalramani and Meegoda 2023; Longendyke et al. 2022; Marsh et al. 2024).

Other damaging technologies that have shown promise in eliminating PFAS compounds and effectively addressed society’s pressing need to clean up this dangerous family of compounds include supercritical water oxidation (Chiang et al. 2023; Krause et al. 2021), plasma (Singh et al. 2021), photocatalysis (Chowdhury et al. 2021; Verma et al. 2024), electrochemical oxidation (Smith et al. 2023), and sonolysis (Gomez-Ruiz et al. 2017; Kewalramani et al. 2022).

Sonolysis, or high-frequency ultrasound, has shown to be an extremely effective technique for mineralization of PFAS in water (Kewalramani et al. 2022, 2023; Kewalramani and Meegoda 2023). Through a process known as acoustic cavitation, this ultrasound produces micro-to nano-sized bubbles (MNBs) when it is applied in a liquid medium. Once these MNBs, which were created during sonic cavitation, grow to a resonant size, they experience adiabatic collapse. When a bubble implodes, the extreme compression causes an adiabatic temperature rise, which raises the temperature to a point where light may be released. The light released during the bubble implosion is a brief flash that lasts for roughly 10 ns (Kewalramani et al. 2022). Many investigations have demonstrated that during the collapse of cavitation bubbles in ultrasound technology, temperatures can reach up to 5000 K, and pressures exceed 500 bars momentarily (Campbell & Hoffmann 2015; Kewalramani et al. 2022). This extreme environment effectively aids in the mineralization of PFAS compounds at ambient temperatures, preventing the release of gaseous fluoro-organics and leading to the formation of fluoride ions and CO_2_, hence offers a promising approach to mineralize PFAS without generating harmful products or emissions (Kewalramani et al. 2023; Kewalramani and Meegoda 2023).

At laboratory bench-scale tests or pilot-field tests, many advanced oxidation treatments that eliminate PFAS have exhibited promise; nevertheless, only a small number of technologies have been proven in the field and are widely acknowledged (Longendyke et al. 2022; McDonough et al. 2022). Treating PFOS and PFOA, the most often found PFAS chemicals in drinking water and groundwater, is the main goal of the majority of PFAS destruction methods (Kewalramani et al. 2023;  Kewalramani and Meegoda 2023; Perera & Meegoda 2024; Simon et al. 2019). Therefore, a significant gap exists in the current research, as there is limited investigation into the effectiveness of PFAS removal in real-world samples with more complex molecular matrices. This highlights the need for further studies that account for the challenges posed by such complex environments in PFAS degradation.

In this study, we look to address the gap in existing research related to the degradation of PFAS in real-world samples with complex chemical matrices. While traditional methods have shown limitations in fully mineralizing highly stable PFAS compounds, particularly in challenging environments like industrial wastewater and aqueous film-forming foam (AFFF), this research explores the use of ultrasound technology to overcome these barriers. By focusing on real waste streams such as groundwater (GW), still bottoms (SB), and film-forming foam (AFFF), this research aims to demonstrate the effectiveness of sonolytic treatment under conditions that more accurately reflect practical applications. This study uses ultrasound-induced cavitation to mineralize PFAS molecules, specifically targeting the strong carbon–fluorine bonds. The extreme temperatures and pressures generated during cavitation enable chemical reactions that are otherwise difficult to achieve. Additionally, the resulting fluoride, sulfate, and chloride ions were measured to quantify mineralization and understand the degradation pathways in these complex matrices. This study offers new insights into the potential of ultrasound as a scalable and efficient technique for PFAS destruction in real environmental settings.

Recent pilot-scale studies have demonstrated that sonochemical oxidation can effectively remove PFAS from aqueous film-forming foam (AFFF), achieving removal efficiencies ranging from 27 to 91.5% while operating at lower energy costs (Gole et al. 2018). However, challenges remain in scaling up sonolysis for full-scale implementation, including high energy consumption, optimization of operating parameters such as ultrasound frequency and power, and integration into existing water treatment infrastructure (Tshangana et al. 2025). For example, one study reported optimal PFAS degradation using 1000-kHz frequency and 400-W power in a resin-regeneration stream, leading to substantial mineralization with some volatile organic fluorides (VOFs) still detected (Fuller et al. 2024). To improve energy efficiency, researchers have recommended reducing power density, optimizing matrix chemistry, and adapting to the pollutant-specific behavior of PFAS under various water conditions (Tshangana et al. 2025). These findings highlight the importance of continued innovation to overcome scale-up barriers and demonstrate the feasibility of ultrasound as a practical solution for PFAS destruction in real-world applications.

## Materials and methods

### Reactor tank

**Fig. 1:**
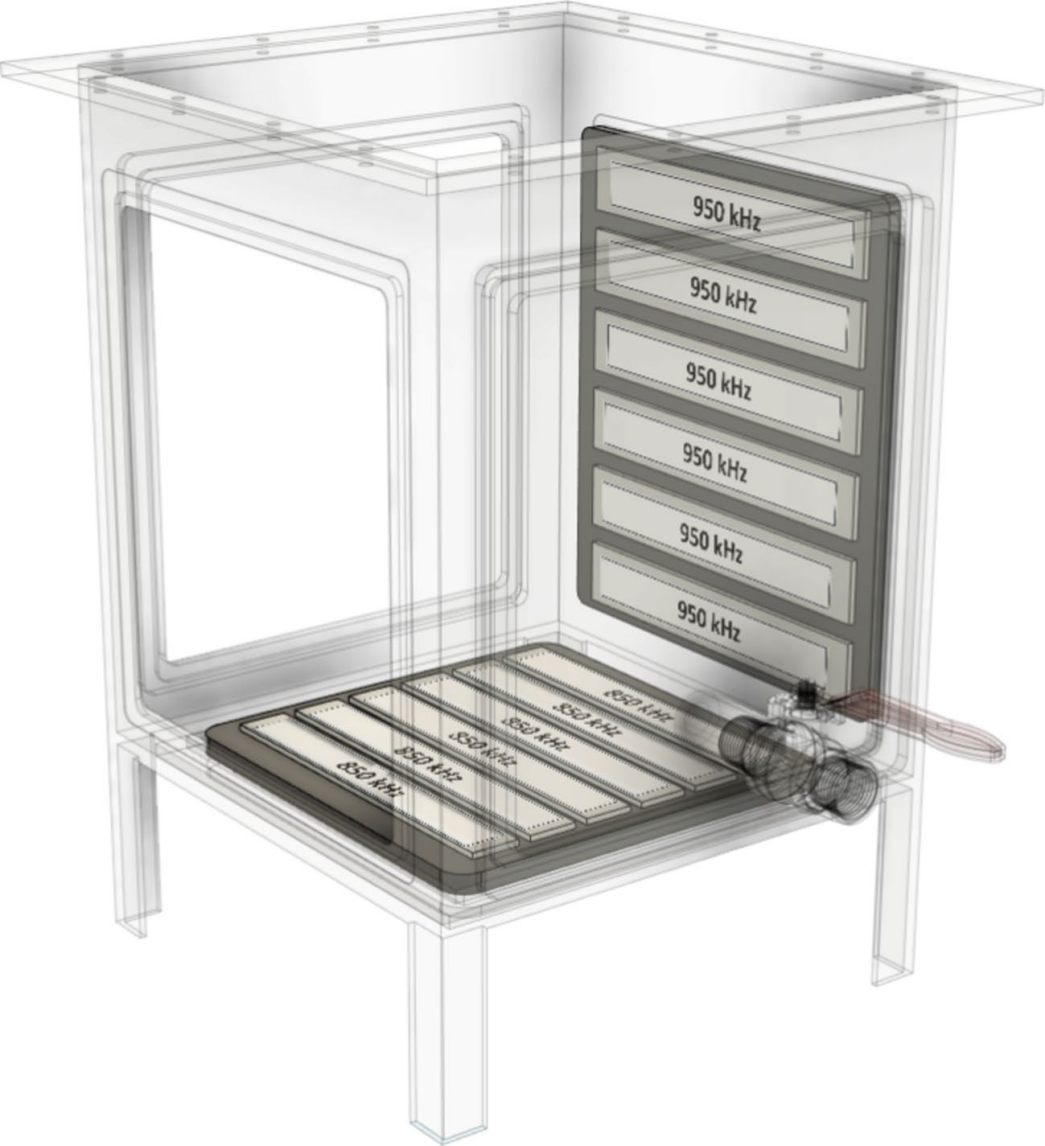
Diagram of transducer configurations in the ultrasound reactor

The plate of the reactor to resonate at 850 kilohertz (kHz) and additional piezoelectric elements were installed on the side plate to resonate at 950 kHz. The reactor was made of electropolished 316 stainless steel and was equipped with cooling coils with a 1/4-in. diameter, which were coupled to a 6-L chiller unit supplied by Vevor (Rancho Cucamonga, California). Two radiofrequency (RF) generators from PCT Systems (part number PCT 6000 series) were used to supply electricity to the reactor’s piezoelectric elements. The power delivery of the RF generator was tuned to the resonant frequency of the crystal arrays within the transducers. The generators were integrated units that both amplified the signal power and produced the desired frequency for the transducers, which were input via the front display of the unit. When operated in multiplex mode, these transducers received electrical power in a sequential fashion. Each array’s piezoelectric elements were powered by the RF generator for a duration of 1 s, after which the process was repeated cyclically for each element. In this study, we used a 10-L reactor tank as illustrated in Fig. 1. The design and operational details of the reactor were described in our previous research (Kewalramani et al. 2023; Kewalramani and Meegoda 2023; Marsh et al. 2024).

### Material

Agilent Milli-Q water (> 18 MΩ·cm) was used in the experiment. Bond Elute PFAS WAX cartridges, purchased from Agilent, were utilized for solid-phase extraction. PFAS standards (USEPA 533) were obtained from Wellington Laboratories, while Thermo Fisher Scientific supplied the total ionic strength adjustment buffer (TISAB II), fluoride with TISAB II standards, ammonium acetate, and HPLC-grade methanol. Additionally, deuterium oxide (D_2_O) solvent and 4′-(trifluoromethoxy) acetanilide were sourced from Sigma-Aldrich.

### Sample information

Three different PFAS waste streams were sent to the New Jersey Institute of Technology for testing: groundwater (GW), still bottom (SB), and aqueous film-forming foam (AFFF). Each waste stream had a distinct PFAS concentration and composition. The total PFAS concentration in GW samples was 0.853 mg/L, with PFOA as the predominant component. In SB samples, total PFAS levels reached 592.9 mg/L, with PFOA accounting for approximately 70% (405 mg/L) of the total PFAS content. AFFF samples contained the highest PFAS concentration, measuring 8738.52 mg/L, with PFOS as the dominant compound, comprising nearly 80% (6802.165 mg/L) of the total PFAS.

To ensure consistency across experiments and avoid matrix-related interference such as excessive foaming or signal saturation during analysis, all raw samples were diluted using Milli-Q water to predefined target concentrations. The final target concentrations were determined based on a combination of factors, including (i) analytical instrument detection limits, (ii) expected ultrasound degradation kinetics from prior work, and (iii) safe operational parameters of the ultrasonic reactor.

The resulting target concentrations were 0.5 mg/L for GW, 12 mg/L and 18 mg/L for SB (representing two dilution sets), and 42 mg/L for AFFF, as shown in Table 1. Additional details on raw sample information and dilutions are provided in Appendix Tables 2, 3, and 4.

**Table 1:** PFAS source, target concentration, and corresponding treatment duration

PFAS source	Target PFAS concentration (mg/L)	Treatment time (hrs)
GW	0.5	6.0
GW	0.5	12.0
SB	18.00	6.0
SB	18.00	8.0
SB	12.00	9.0
SB	12.00	12.0
AFFF	42.00	6.0
AFFF	42.00	12.0

### Experimental procedures and sample collection

During the treatment of PFAS waste solutions, cooling water was cycled through the reactor’s cooling coil in order to efficiently manage the heat produced during sonication. The sonication procedure was carried out in cycles of 15 min. For every cycle, the transducers were powered on for 15 min, and then the piezo crystals were allowed to cool and release the heat they had absorbed for another 15 min. This technique ensured that the system ran between 20 and 30 °C, which is the appropriate temperature range for the piezo crystals and the PFAS waste that was being treated. The PFAS source and target concentration determined the different treatment timeframes. GW samples with a target PFAS concentration of 0.5 mg/L were treated for 6 and 12 h. SB samples with target concentrations of 18 mg/L were treated for 6 and 8 h, while those with 12 mg/L were treated for 9 and 12 h. AFFF solutions with a concentration of 42 mg/L were treated for 6 and 12 h. Treatment durations were selected based on preliminary optimization experiments and past studies indicating that degradation plateaus after 12 h. The range of timeframes (6 to 12 h) allowed for comparison between short-term and extended sonolytic effects across sample types and concentrations. Across all tests, the power density was consistently maintained at 118 W/L, while the sample volume remained fixed at 10 L. This rigorous standardization provided a controlled experimental environment, ensuring accurate and reliable comparisons of PFAS mineralization within the waste matrix. All degradation results are reported as mean ± standard deviation (SD) based on two replicates per condition.

Sample collection was done on a regular basis during the trials. Using a disposable dropper, 5 mL of samples were taken every 30 min from the reactor’s top for the determination of fluoride ions and PFAS concentrations. The samples were then transferred to polypropylene vials for storage. Additionally, to determine the total organic content of PFAS, 250 mL samples were collected through the sampling port and placed in an HDPE bottle both before and after each experiment. The samples were then processed using a solid-phase extraction procedure in accordance with USEPA procedure 533 method (Wendelken 2018). A 100 mL primary sample was collected at the beginning and end of each trial in a 100-mL HDPE bottle for chemical oxygen demand (COD) analysis. Additionally, 50 mL samples were taken from the sample port in polypropylene vials, both before and after treatment, separately for inorganic anion analysis and metal analysis. Before being analyzed, triplicate samples were obtained and kept at 4 °C inside a refrigerator. After each test, the reactor was completely cleaned three times with tap water, then twice with Milli-Q water, and finally rinsed with a mixture of methanol and Milli-Q water (70:30 v/v) to prevent cross-contamination between tests (Kewalramani et al. 2023).

### PFAS analysis

PFAS analysis (25 PFAS analyte-List of method USEPA 533) of liquid samples was carried out using liquid chromatography with tandem mass spectrometry (Agilent 6470 Triple Quadrupole LC/MS System) utilizing the modified USEPA 533. An aliquot of the sample was diluted with methanol at the proper ratio to obtain a final concentration of less than 100 parts per billion. The diluted samples were then filtered through a 0.25-μm polyethersulfone syringe filter for LC/QQQ analysis (Kewalramani et al. 2022, 2023; Kewalramani and Meegoda 2023; Marsh et al. 2024).

Degradation efficiency (DE) calculated using Eq. 1, serves as a metric to evaluate the effectiveness of the treatment (Awoyemi et al. 2024; Chen et al. 2024). In the context of PFAS treatment, DE denotes the degree to which these compounds are degraded or converted into less hazardous substances during a remediation procedure. Typically, DE is assessed by measuring the decrease in total PFAS concentrations. High DE indicates an effective treatment that can reduce the environmental and health risks associated with PFAS contamination. Liquid chromatography-mass spectrometry (LC–MS) data was utilized in this study to compute the DE.

1 $$\mathrm{DE}\; \left(\%\right)=\left(1- \frac{{C}_{\mathrm{f}}}{{C}_{\mathrm{o}}}\right)\times 100$$

where:

- *C*_o_ = initial concentration of PFAS before treatment.
- *C*_f_ = final concentration of PFAS after treatment.

Moreover, the degradation kinetics of PFAS were analyzed assuming pseudo-first-order reaction kinetics, using the model:

2 $$\mathrm{ln}(\frac{C}{{C}_{\mathrm{o}}})=-kt$$

where *C* is the PFAS concentration at time *t*, *C*_*0*_ is the initial concentration, and *k* is the pseudo-first-order rate constant.

### Anion analysis

Suppressed conductivity ion chromatography (IC), employing a Dionex IC-1500 system with a Dionex Ion Pac AS 18 analytical column (4*250 milimeters [mm]) and AG 18 guard column (4*50 mm), was used to analyze anions concentrations (fluoride and sulfate). A 17 mM of injectable eluent (KOH) was used for 6 min.

### Nuclear magnetic resonance (NMR) analysis

Using solid phase extraction, the 250 mL of samples collected at the start and finish of the trials were concentrated to 5 mL of methanol for analysis by 19 F NMR (Kewalramani et al. 2022, 2023; Marsh et al. 2024). The sample pH was corrected to 6.5 ± 0.5 before being concentrated using the modified USEPA 533 technique into 5 mL of 1% methanolic ammonium hydroxide (Wendelken 2018). For every extracted sample, 4′-(trifluoromethoxy) acetanilide was added as an internal standard. For analysis, 0.6 mL of a 1:9 D_2_O solvent and sample extract solution was put in a glass tube. With a spectrum breadth of − 220 to 20 mg/L, the 19 F NMR spectra were acquired with a Bruker AV300 MHz NMR spectrophotometer.

### Fluoride ion analysis

Using a fluoride-ion selective electrode (F-ISE) from Thermo-scientific, which monitors the activity of fluoride ions as a voltage response (Kewalramani et al. 2022, 2023; Kewalramani and Meegoda 2023; Marsh et al. 2024), the concentration of inorganic fluoride was determined. The aliquot sample was diluted 1:1 with TISAB II in order to modify the solution’s ionic strength.

### Metal analysis

Metal concentrations, including chromium (Cr), iron (Fe), manganese (Mn), and calcium (Ca), were measured using inductively coupled plasma mass spectrometry (ICP-MS) (Agilent 7900). Samples were collected in 50-mL polypropylene vials, filtered through 0.45-μm polyethersulfone syringe filters. All measurements were conducted under controlled conditions to ensure accuracy and reproducibility.

## Test results

### PFAS degradation

**Fig. 2:**
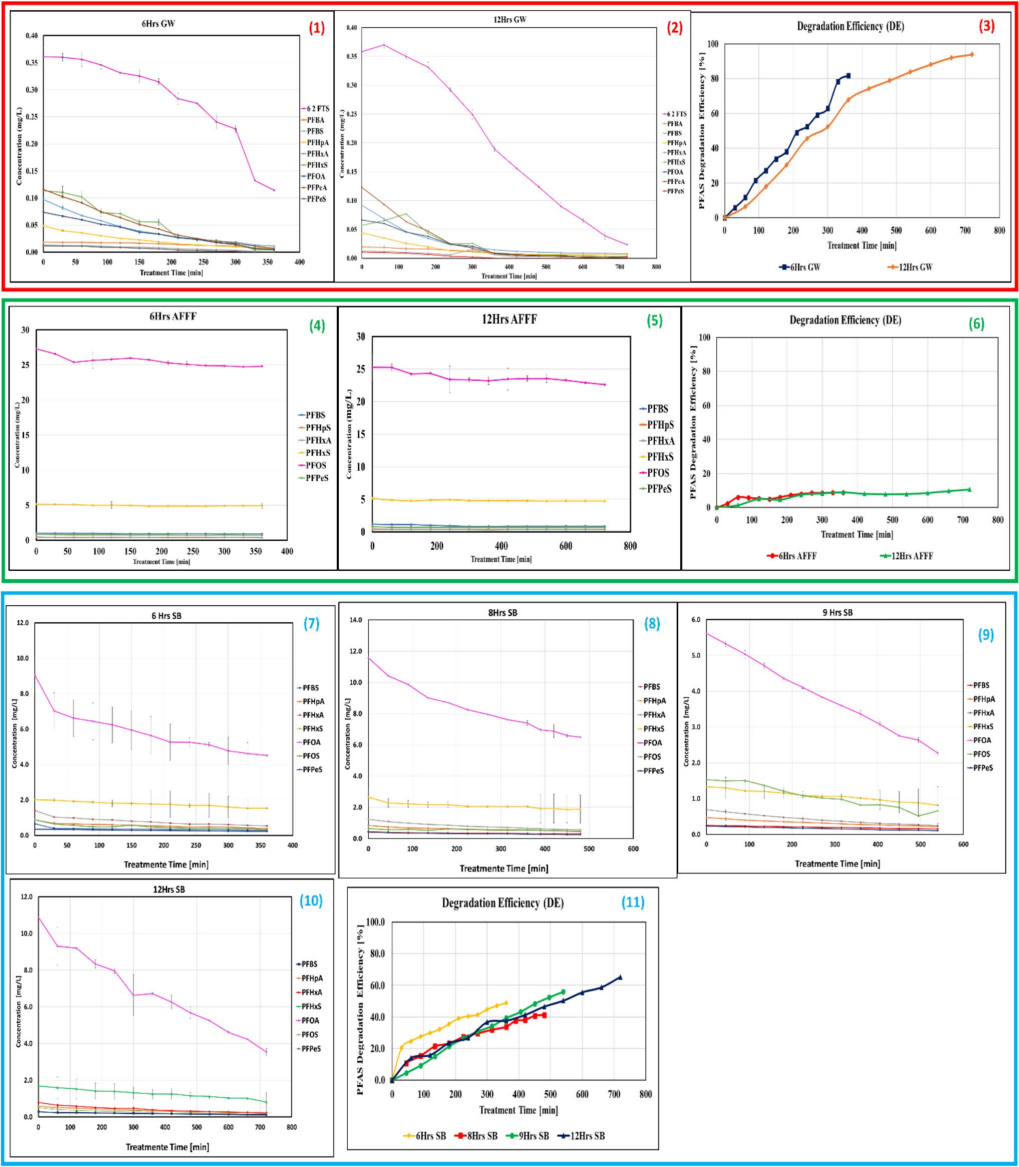
PFAS concentration and degradation across different matrices (GW, SB, and AFFF) under various treatment durations. Error bars represent standard deviation (SD) from duplicate measurements, where applicable. For datasets based where SD was negligible, error bars may not be visible

**Fig. 3:**
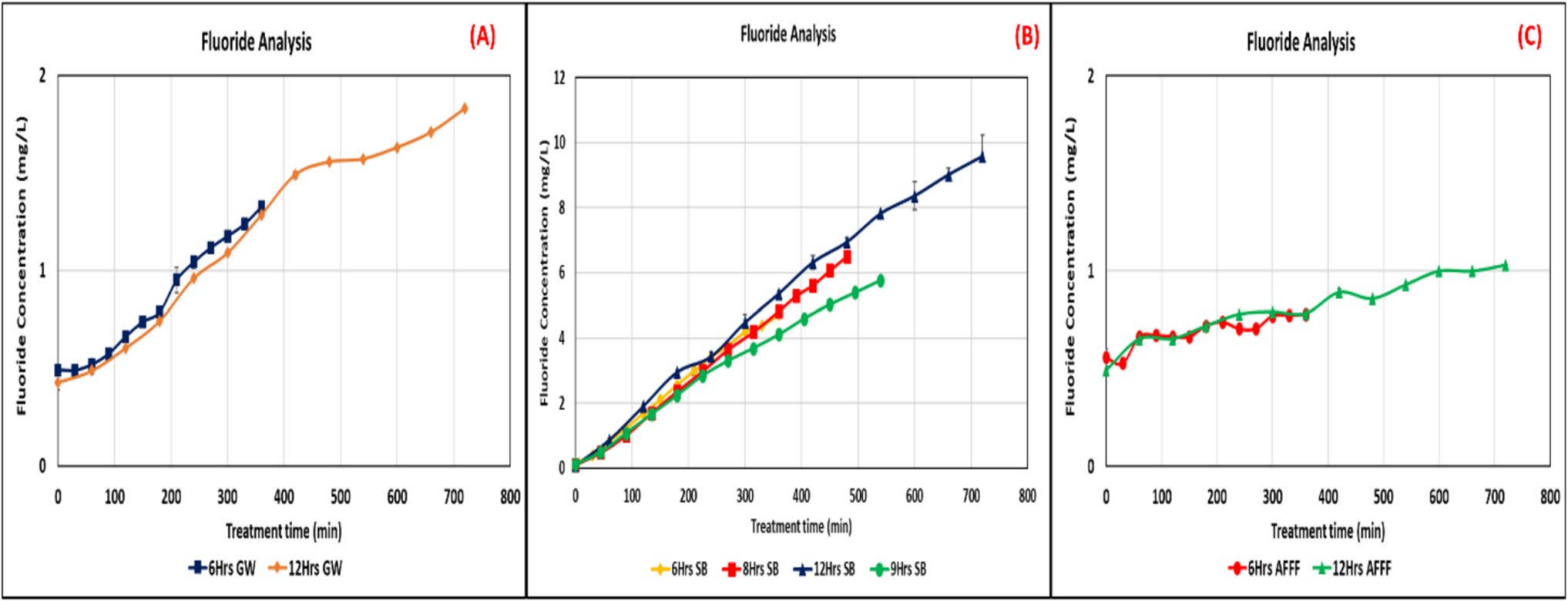
Fluoride concentration vs time. **A** GW, **B** SB, and **C** AFFF samples. Error bars represent standard deviation (SD) from duplicate measurements, where applicable. For datasets based where SD was negligible, error bars may not be visible

The degradation of PFAS through ultrasound treatment was evaluated in three distinct environmental matrices: GW, SB, AFFF waste samples. The primary goal was to assess the impact of matrix composition on DE over treatment durations of 6, 8, 9, and 12 h. The degradation process was analyzed using LC–MS for PFAS concentration reduction and a fluoride ion-selective electrode (F-ISE) to quantify defluorination. The LC–MS results (Fig. 2 (1) and (2)) reveal that both 6-h and 12-h ultrasound treatments led to significant degradation of PFAS in GW samples. Initially, the degradation rate was steady, particularly during the first 300 min. However, in the extended 12-h treatment, the degradation curve plateaued, suggesting that equilibrium was approached. Among the PFAS present, 6:2 FTS exhibited the highest degradation efficiency, followed by PFPeA, which also showed significant degradation. The F-ISE results (Fig. 3a) corroborate these findings, demonstrating that fluoride ion release increased over time, further confirming PFAS mineralization. However, fluoride concentrations were lower in GW samples than in SB samples, indicating a lower overall PFAS load in GW.

The SB samples exhibited a similar degradation pattern, as shown in Fig. 2 (7) to (10). The 6-h treatment achieved approximately 40% DE within the first 200 min, leveling off around 49% by the end. The 12-h treatment, in contrast, demonstrated the highest DE, exceeding 65% by the end of the experiment. The consistent trend across different treatment durations indicates that prolonged sonolysis enhances mineralization. Among the PFAS compounds present in SB, PFOA exhibited the highest degradation efficiency.

To be more specific, long-chain PFAS degrades more efficiently than short-chain counterparts. This is attributed to the higher hydrophobicity of long-chain PFAS, which facilitates their accumulation at the cavitation bubble interface, where high-temperature pyrolysis occurs. Short-chain PFAS exhibit lower affinity for these sites, leading to reduced degradation efficiency, with PFBA showing the slowest degradation rate and the highest resistance to defluorination (Campbell & Hoffmann 2015; Cheng et al. 2008; Kewalramani et al. 2023; Kewalramani and Meegoda 2023; Marsh et al. 2024). Moreover, for the same number of carbon atoms, carboxylates broke down more quickly than sulfonates (Campbell & Hoffmann 2015; Fernandez et al. 2016). Because carboxylates have lower thermal activation energies, there is a greater rate of carboxylate breakdown (Bentel et al. 2019; Psillakis et al. 2009).

Fluoride release (Fig. 3c) showed a proportional increase with treatment duration, confirming continuous defluorination. The increased fluoride concentration in SB samples compared to GW suggests that PFAS degradation was more substantial due to the higher initial PFAS concentrations in SB.

The AFFF samples, containing high PFOS concentrations, exhibited minimal degradation (Fig. 2 (4) and (5)). Both 6-h and 12-h treatments showed notably low DE, primarily attributed to the extreme resistance of PFOS to sonolysis. A foam layer formed at the liquid surface, which likely impeded ultrasonic wave penetration, reducing the effectiveness of degradation. The F-ISE results (Fig. 3b) further illustrate that fluoride release was negligible, reinforcing the conclusion that PFAS mineralization in AFFF samples was limited. The persistent nature of PFOS and its strong C–F bonds make it challenging to break down under the current treatment conditions.

Overall, the findings imply that the hydrophilic functional group has accelerated the breakdown of PFAS (Kewalramani et al. 2023). As the initial PFAS concentration increased, the fluoride release concentration also showed a proportional rise. A similar trend was observed by Fernandez et al. (2016); Kewalramani et al. (2023); and Shende et al. (2019). However, when the concentration exceeded 18 mg/L, the rate of fluoride formation reached a steady state, showing no further significant increase. This pattern was also noted by previous research (Kewalramani et al. 2023; Kewalramani and Meegoda 2023); Rodriguez-Freire et al. 2016). It was ascribed to the PFAS adsorption rate-limiting step at the bubble interface, where PFAS saturation of the cavitational bubble interface is proposed at high starting concentrations (Cheng et al. 2008; Rodriguez-Freire et al. 2016; Shende et al. 2021). At PFAS concentrations exceeding the cavitation bubble’s saturation limit, excessive foaming occurred during ultrasonic treatment. This negatively impacted the process, leading to reduced fluoride formation rates at the highest initial concentration (Marsh et al. 2024; Rodriguez-Freire et al. 2015).

The degradation of PFAS compounds was monitored over a 6-h treatment period, and the concentration data were fitted using a pseudo-first-order kinetic model to evaluate degradation rates. Appendix Table (5) presents the calculated rate constants (*k*, h^-^1), corresponding half-lives (*t*_1_/_2_*,* h), and coefficients of determination (*R*^2^) for each compound across three matrices: groundwater (GW), AFFF-contaminated water (AFFF), and synthetic background water (SB). In general, PFAS compounds in the GW matrix exhibited higher rate constants (e.g., PFHxS: *k* = 0.5518 h^-^1, *t*_1_/_2_ = 1.26 h, *R*^2^ = 0.91), suggesting more efficient degradation compared to the AFFF matrix, where degradation was significantly slower (e.g., PFHxA: *k* = 0.0013 h^-^1, *t*_1_/_2_ = 533.08 h, *R*^2^ = 0.0156). This difference may be attributed to the higher complexity and organic load in AFFF, which likely inhibits the generation or effectiveness of reactive species during sonolysis.

In the SB matrix, moderate degradation rates were observed. Notably, PFOS and PFPeS showed relatively high rate constants (PFOS: *k* = 0.1254 h^-^1, PFPeS: *k* = 0.0738 h^-^1), while PFHpA and PFHxA degraded slower (*k* = 0.0628 and 0.1358 h^-^1, respectively), but with strong correlation (*R*^2^ > 0.87). These findings suggest that chain length, head group (sulfonate vs carboxylate), and solution composition all influence the degradation kinetics. Most fits showed strong correlation (*R*^2^ > 0.85), validating the suitability of pseudo-first-order modeling. Exceptions were observed for some AFFF cases (e.g., PFHxA), where poor fit (*R*^2^ = 0.0156) indicated potential interference or nonlinear degradation behavior. These results enable direct comparison of degradation efficiency across compounds and matrices and support the utility of ultrasound-based treatments under varied environmental conditions.

### Anion analysis

The release of fluoride ions is critical in determining the mineralization of PFAS compounds, particularly through the cleavage of C–F bonds during sonolytic treatment. According to the Table (6) in the Appendix, the SB samples showed a noticeable increase in fluoride concentration after treatment, especially in the 12-h (12-h SB), where fluoride concentration increased from 0 to 8.71 mg/L. This indicates that sonolysis was able to achieve significant defluorination, although the initial concentration of fluoride was zero before treatment.

For the GW samples, fluoride concentrations increased slightly after treatment, from 0.54 to 1.39 mg/L after 6-h treatment and from 0.51 to 1.78 mg/L after 12-h treatment. This suggests that defluorination occurred at a slower rate for GW samples, due to lower PFAS concentrations. For the AFFF samples, which contain PFOS, fluoride release was minimal, with an increase from 0.75 to 1.03 mg/L for the 6-h treatment and from 0.64 to 1.37 mg/L for the 12-h treatment. These results confirm that PFOS, a key component in AFFF, is highly resistant to degradation under the sonolysis conditions used, and limited defluorination was achieved. Hence the released energy from sonolysis was used to oxidize other organic compounds in the AFFF sample.

The sulfate concentration of the SB samples reduced from 842.53 to 363.96 mg/L, 449.97 to 371.01 mg/L, 298.31 to 247.91 mg/L and 347.39 to 255.74 mg/L when treating the samples for 6, 8, 9, and 12 h respectively. Initial high sulfate concentration of the SB samples denotes that during distillation or evaporation process of SB samples, non-volatile compounds like sulfates concentrate as water and volatile components are removed. And with sonolytic treatment, the sulfate concentration has been reduced. Interestingly, in both the GW and AFFF samples, the initial sulfate concentration was 0 mg/L, and it remained unchanged after treatment, implying that sulfate containing PFAS compounds were not present in significant quantities in these samples.

Chloride concentration is an indirect measure of possible matrix interactions during treatment, as PFAS compounds do not typically contain chlorine. The SB samples showed a notable decrease in chloride concentration after treatment, particularly in the 6-h SB samples, where chloride levels dropped from 6958.35 to 4026.44 mg/L. This reduction could suggest that the treatment process affected chloride-containing components in the sample matrix, possibly through interactions with the reactor setup or through unrelated to PFAS degradation.

For the GW samples, chloride concentrations were very low and showed minimal change, with values increasing slightly from 3.63 to 3.67 mg/L after the 6-h treatment. This suggests that chloride was not a significant factor in the degradation of PFAS in GW samples. In the AFFF samples, chloride levels before and after treatment were 0 mg/L, indicating that chloride was not involved in the AFFF matrix or its degradation.

The data indicate that fluoride release was modest across all sample types, reflecting the high resistance of PFAS compounds, particularly PFOS, to degradation under sonolytic conditions. Sulfate reduction was more prominent in the SB samples, suggesting partial degradation of sulfonated PFAS compounds. Meanwhile, chloride reduction was observed primarily in SB samples, likely indicating matrix interactions rather than direct PFAS degradation. The findings point to the need for more aggressive treatment methods or extended treatment times to enhance defluorination, particularly for resistant compounds like PFOS in AFFF samples.

### NMR and total amount of organically bound fluorine (TOF) analysis

**Fig. 4:**
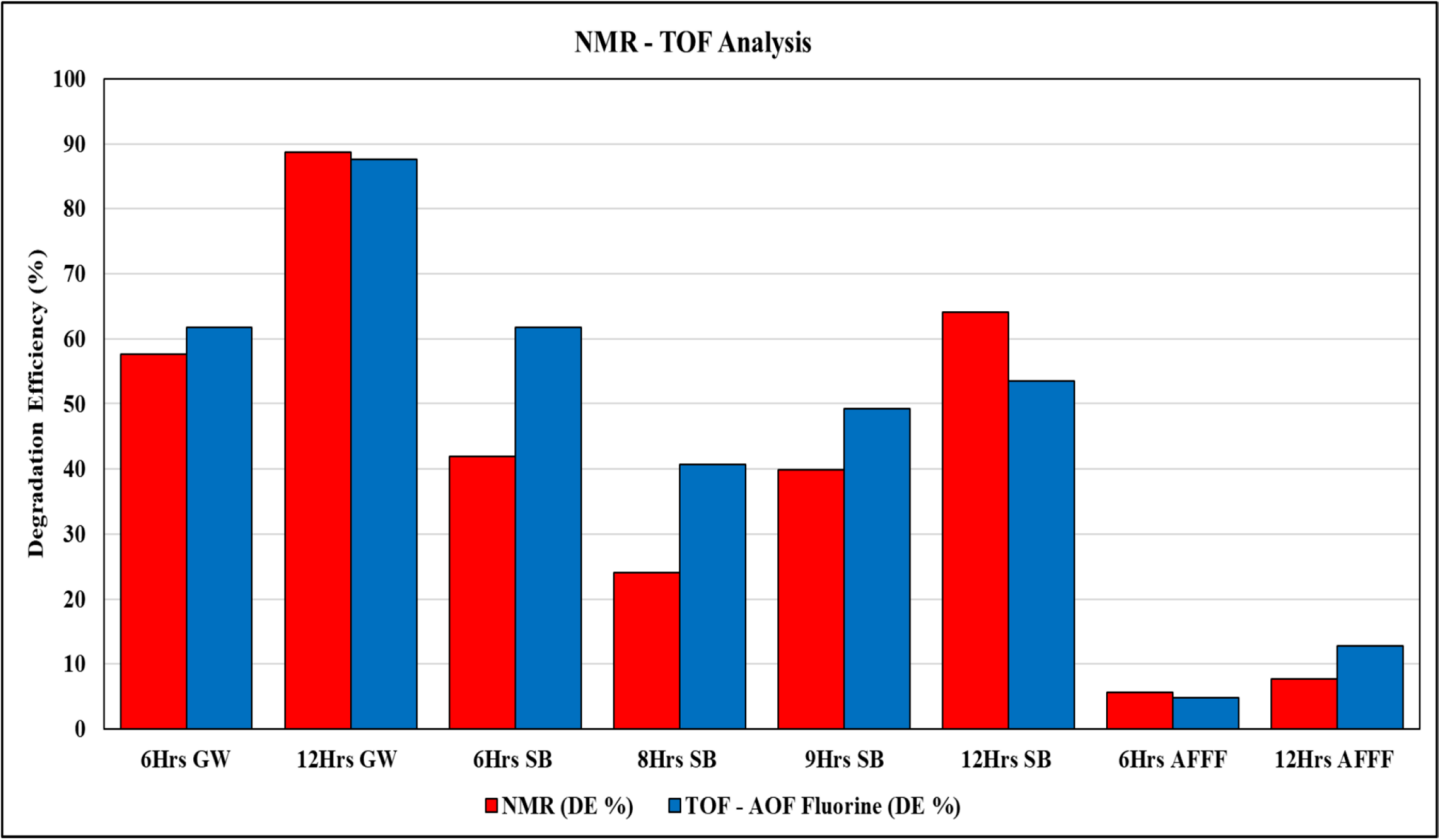
Degradation efficiency of PFAS via NMR and TOF analysis in various sample matrices: groundwater, still bottom, and AFFF samples

To assess PFAS degradation, NMR and TOF-adsorbable organically bound fluorine (AOF) analyses were performed, providing complementary insights into structural decomposition and fluoride release (Fig. 4). NMR spectroscopy is a non-targeted analytical technique capable of detecting multiple PFAS species based on their fluorine environments, including terminal –CF_3_ groups, while TOF-AOF quantifies the total fluoride release, indicating mineralization. The combination of NMR and TOF-AOF analyses provides a comprehensive understanding of PFAS transformation pathways and highlights the potential for optimizing sonolytic treatment for complex environmental matrices.

GW samples showed significantly higher degradation compared to SB and AFFF matrices. The 6-h GW samples demonstrated a DE of 57.7% (NMR) and 61.76% (TOF-AOF), indicating substantial PFAS breakdown. The –CF_3_ concentration in NMR results decreased from 0.000198 mM before treatment to 0.000084 mM after treatment, corroborating the structural breakdown of PFAS compounds. The most effective treatment was observed in the 12-h GW samples, where NMR and TOF-AOF analyses closely aligned at 88.8% and 87.64%, respectively. The –CF_3_ concentration from the NMR results dropped from 0.000236 to 0.000026 mM (Appendix Table 7), reinforcing the near-complete degradation and mineralization of PFAS. A decrease in –CF_3_ concentration was also observed in other studies after PFAS degradation, where mineralization efficiency depends on structural characteristics, treatment duration, and matrix composition (Kewalramani et al. 2023; Kewalramani and Meegoda 2023; Marsh et al. 2024).

For the SB samples, degradation efficiency (DE) varied with treatment duration. The 6-h SB samples exhibited a moderate DE of 41.9% via NMR, while TOF-AOF recorded a higher DE of 61.76%, suggesting partial defluorination and breakdown of PFAS structures. However, at 8 h, DE decreased to 24.1% (NMR) and 40.74% (TOF-AOF), indicating potential intermediate formation or slower degradation kinetics. By 12 h, NMR detected an increase in DE to 64.2%, reflecting enhanced PFAS transformation, while TOF-AOF recorded 53.52%, suggesting incomplete defluorination relative to structural degradation. The presence of distinct –CF_3_ concertation in NMR result, as shown in Appendix Table 7, indicates that although degradation occurred, residual fluorinated species persisted, particularly in shorter treatment durations. Conversely, AFFF samples exhibited the lowest degradation across all matrices, likely due to the high PFOS content and its well-documented resistance to sonochemical treatment. For the 6-h AFFF samples, NMR indicated a minimal DE of 5.6%, with TOF-AOF measuring a comparable 4.87%, signifying limited defluorination. The 12-h AFFF samples displayed only slight improvements, with NMR at 7.7% and TOF-AOF at 12.8%, reaffirming the challenges in degrading PFOS-rich matrices. The persistent –CF_3_ concentration in NMR results indicate that despite prolonged treatment, the core structure of PFOS remains largely intact, emphasizing the need for alternative or enhanced degradation strategies.

To better understand the extent of PFAS mineralization, a full fluoride mass balance was conducted by comparing TOF results before and after treatment with the amount of fluoride ion (F^-^) released (Appendix Table 8). The results demonstrate that GW samples exhibited the most complete mineralization. After 12 h of treatment, 87.65% of organo-fluorine was removed, with 81.76% converted to F^-^, indicating minimal intermediate accumulation. In contrast, SB samples showed high defluorination (up to 64.23%) but relatively low fluoride yield (as low as 22.22%), suggesting the formation of fluorinated by-products not captured by F^-^ measurement. The largest gap between defluorination and fluoride yield was observed in SB samples treated for 6 h, where only 25.62% of the cleaved fluorine was recovered as fluoride, and 12.29 mg/L remained unaccounted. AFFF samples, dominated by PFOS, were the most resistant, with minimal defluorination (13.17%) and only 2.51% fluoride yield, likely due to foam formation and strong C–F bonds in PFOS that inhibit cavitation and radical access. These findings highlight the matrix-specific nature of PFAS degradation under sonolysis. While GW facilitates effective mineralization, high organic loads in SB and surfactant-rich AFFF can inhibit fluoride release and lead to intermediate accumulation. Further screening of degradation by-products using high-resolution mass spectrometry is recommended to fully close the fluorine mass balance and assess the formation of partially fluorinated compounds.

### Metal analysis

**Fig. 5:**
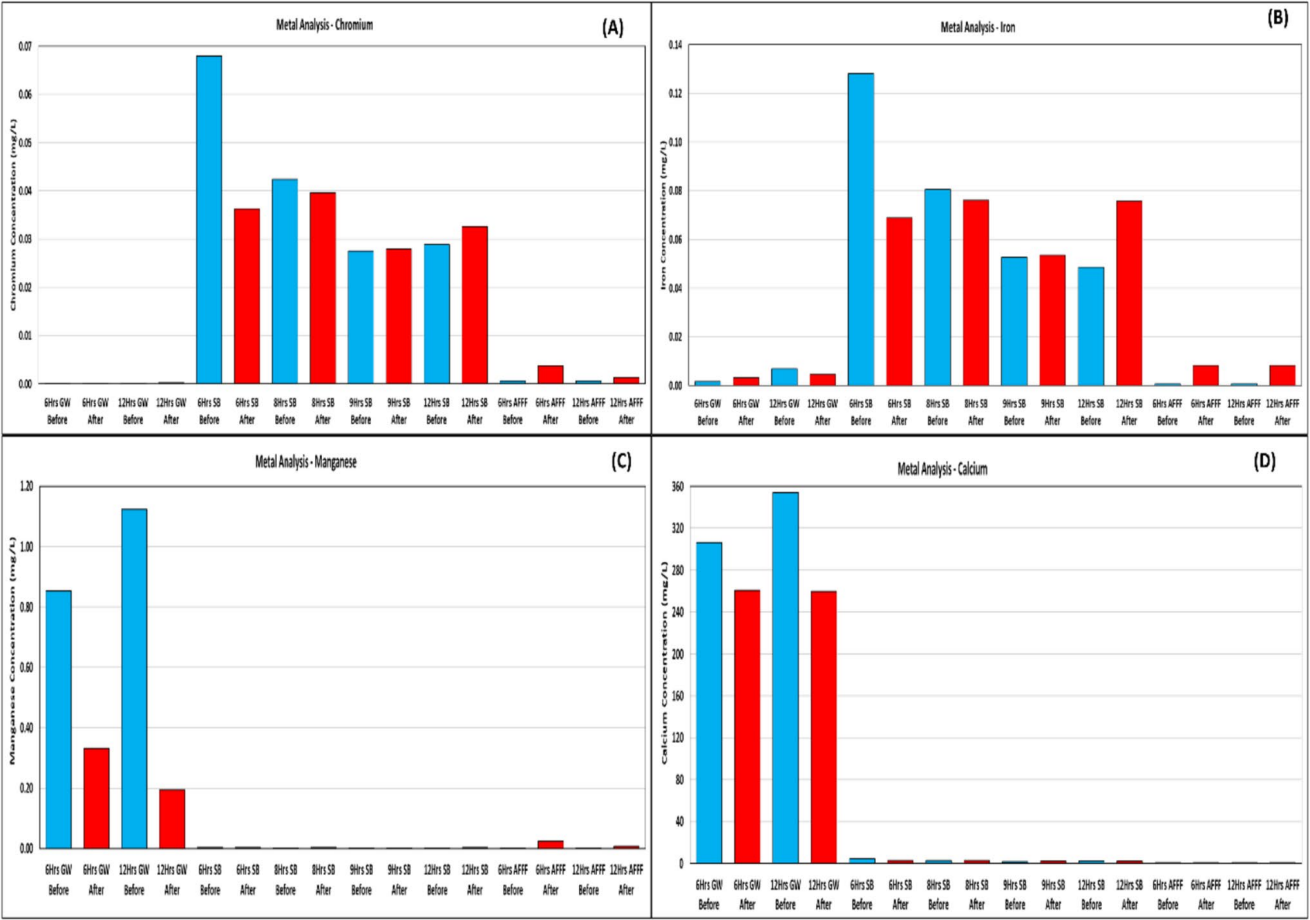
Metal analysis before and after sonolytic treatment: chromium (**A**), iron (**B**), manganese (**C**), and calcium (**D**) concentrations in various sample matrices

Figure 5 illustrates the concentrations of four soluble metal ions, chromium, iron, manganese, and calcium before and after sonolytic treatment across different sample matrices (SB, GW, and AFFF). The data indicate a general decrease in soluble metal concentrations post-treatment, suggesting transformation or removal through sonication. For each metal, the figure highlights unique behavior. In SB samples, there was a significant drop in chromium concentration after ultrasound treatment, particularly for the 6-h treatment, where chromium levels dropped from 0.065 to 0.035 mg/L. For the GW and AFFF samples, chromium levels were initially low and remained relatively unchanged, indicating that chromium played a minimal role in PFAS degradation for these matrices under ultrasonic treatment.

Iron concentrations decreased notably in post-treatment SB samples (e.g., 6-h SB: 0.12 to 0.07 mg/L), though some rebound was observed after longer durations (e.g., 12-h SB), suggesting complex formation. In GW and AFFF samples, iron levels remained mostly unchanged, indicating its minor role in PFAS degradation under sonolysis. This is consistent with findings that ultrasound-assisted metal leaching can selectively dissolve and transform metals through cavitation-driven mechanisms, particularly in acidic environments (Zhang et al. 2013). For GW samples, manganese concentration significantly decreased after ultrasound treatment, particularly in the 12-h GW samples, where it dropped from 1.1 to 0.6 mg/L. In contrast, for SB and AFFF samples, manganese levels were initially low, and no significant changes were observed. The role of manganese in PFAS degradation remains unclear, as previous studies have shown that permanganate has limited efficacy in breaking down PFOS, while ferrate oxidation appears to be more effective (McBeath & Graham 2021). However, manganese compounds, such as permanganate, have been explored as potential oxidants in advanced water treatment processes, though their impact on PFAS solubility and transformation is still debated (McBeath & Graham 2021).

For GW samples, the observed decrease in calcium levels after treatment suggests that calcium might be interacting with either PFAS molecules or degradation byproducts during cavitation. While calcium itself is not directly involved in the chemical degradation of PFAS, its role could involve modifying solution dynamics, potentially facilitating the physical breakdown of PFAS molecules. During bubble implosions, the intense localized heat and pressure may cause calcium to interact with dissociated ions or degradation fragments, influencing the solubility of these species or promoting the formation of insoluble calcium salts (e.g., calcium fluoride or calcium carbonate). This could lead to precipitation, which would account for the drop in measured calcium concentration (Zhang et al. 2013). The formation of calcium fluoride (CaF_2_) has been previously reported as a mechanism for removing fluoride ions released during PFAS degradation, further supporting this observation (Wang et al. 2015). In contrast, for SB and AFFF samples, minimal changes in calcium concentration suggest that calcium did not play a significant role in PFAS degradation or interaction within these matrices. This might be due to different chemical environments in SB and AFFF compared to GW. For instance, SB might already be saturated with certain ions, limiting calcium’s ability to form new complexes or precipitates. AFFF, containing surfactants and other stabilizing agents, could inhibit calcium-PFAS interactions, preventing significant changes in calcium levels. As a result, calcium remains largely unaffected by sonolytic treatment in these samples.

## Effects of water matrix on PFAS treatment degradation

**Fig. 6:**
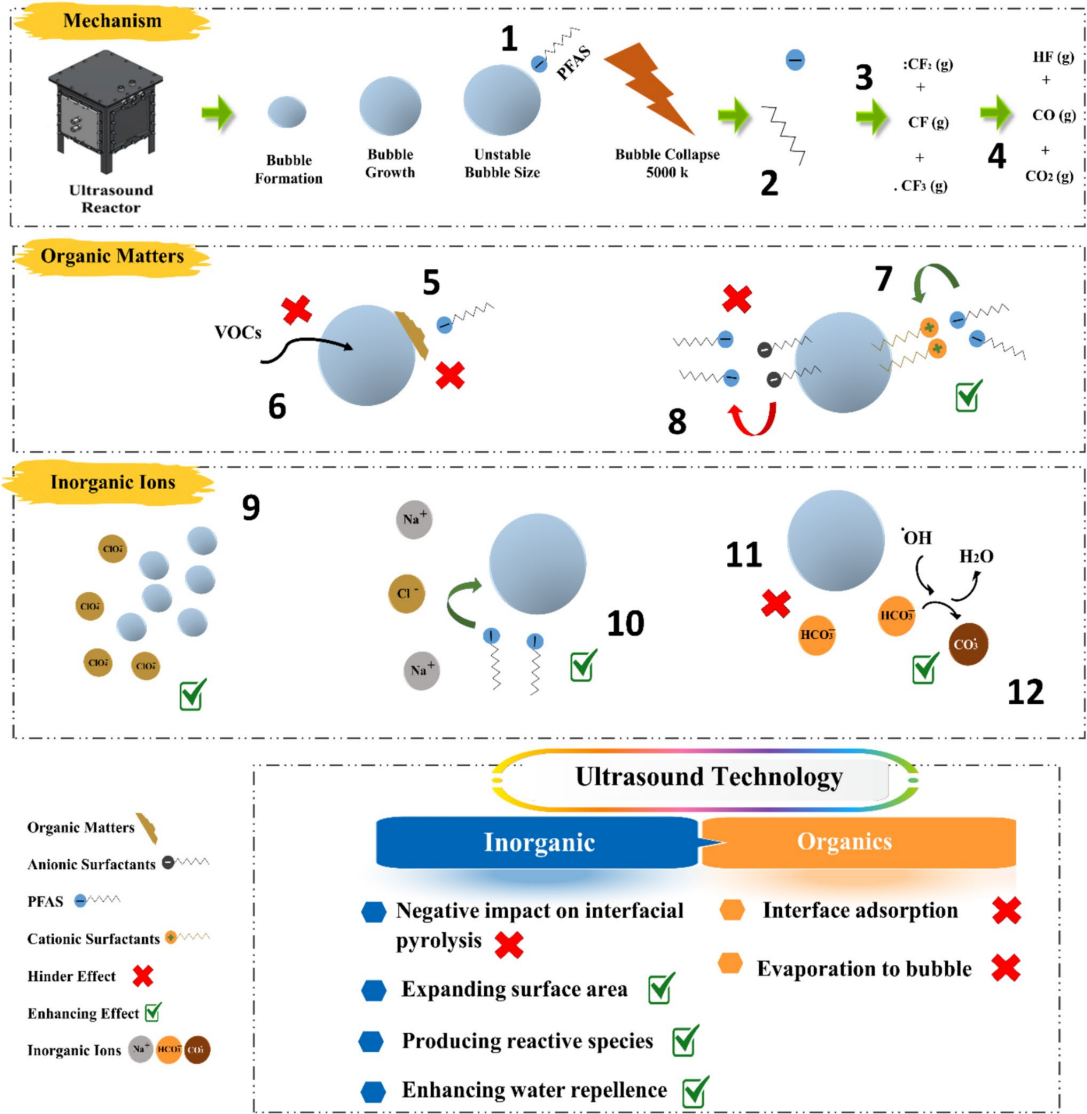
Mechanisms and effects of ultrasound technology on PFAS degradation and interaction with organic matter and inorganic ions

Sonolysis utilizes ultrasound to generate and implode nano/micro bubbles, creating extreme conditions that produce reactive hydroxyl radicals (•OH) and hydrogen radicals (H•) radicals (Eq. 3). PFAS, due to their high surface activity, accumulate at the bubble-water interface, where temperatures reach ~ 5000 K during collapse, leading to pyrolysis (Eq. 4). Water solutes influence the bubble-water interface area, interfacial temperature, and PFAS concentration, all of which are key factors in PFAS degradation (Fig. 6 (1–4)) (Zeidabadi et al. 2023).

3 $${\mathrm{H}}_{2}\mathrm{ O} \mathrm{Ultrasound}\to {}^{\bullet }\mathrm{OH}+{}^{\bullet }\mathrm{H}$$

4 $${C}_{n}{F}_{2n+1}CO{O}^{-}or{ C}_{n}{F}_{2n+1}S{O}_{3}^{-}Ultrasound\to C{ }_{n}{F}_{2n+1}+CO{O}^{-}or S{O}_{3}^{-}$$

The composition of the water matrix plays a significant role in PFAS degradation by sonolysis. Factors such as organic content, dissolved inorganic ions, and surfactant concentrations influence the efficiency of ultrasonic treatment (Zeidabadi et al. 2023). This section focuses on the effects observed in the three sample matrices used in this study: groundwater (GW), still bottom (SB), and aqueous film-forming foam (AFFF).

**Fig. 7:**
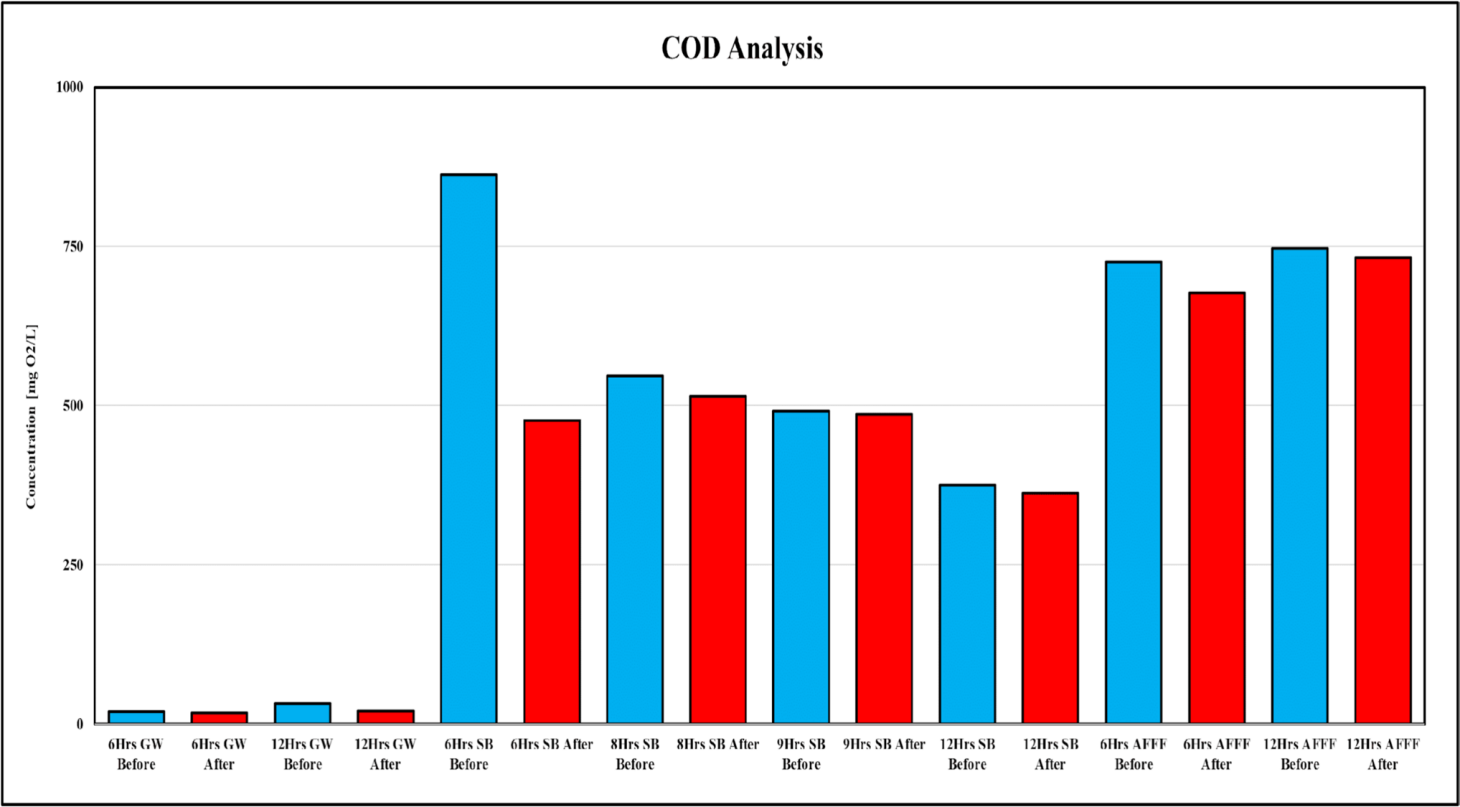
Impact of sonolysis on chemical oxygen demand (COD) across various PFAS-contaminated matrices

The COD results serve as a critical indicator of the organic load and oxidative demand (Kayaalp et al. 2010) in the studied matrices, providing insight into the interactions and potential interference of organic materials with PFAS degradation. Figure 7 shows a significant variation in COD levels before and after sonolytic treatment, highlighting the complex dynamics between organic matter and the degradation process.

### Organic components

In GW samples, the initial COD levels were relatively low, consistent with the simpler composition of this matrix compared to SB and AFFF. Post-treatment COD values exhibited minimal changes, aligning with the moderate defluorination and degradation efficiency observed. The lower organic load likely enabled more direct interactions between PFAS molecules and reactive radicals, resulting in a higher mineralization efficiency compared to the organic-rich SB. The reduced competition for reactive radicals in GW allowed for more effective PFAS breakdown, leading to a more efficient defluorination process. This aligns with previous studies demonstrating that the presence of organic elements in groundwater, such as volatile organic compounds (VOCs), can influence PFAS degradation. VOCs, by evaporating into the bubble vapor phase and adsorbing onto the bubble-water interface, reduce interfacial temperatures and hinder sonolysis efficiency (Cheng et al. 2008). Additionally, the breakdown rates of PFOA and PFOS in groundwater were reported to be 2.3 and 2.6 times slower than those in Milli-Q water, indicating that organic constituents influence sonolytic degradation rates (Fig. 6 (5–6)) (Cheng et al. 2008).

In contrast, SB samples exhibited substantially high initial COD levels, reflecting the dense organic and inorganic constituents present in this waste stream. Post-treatment results demonstrated a consistent reduction in COD across all durations (6, 8, 9, and 12 h), indicating that sonolysis effectively reduced the organic load. This reduction correlated with the release of fluoride ions, suggesting that the breakdown of organofluorine compounds significantly contributed to the decrease in COD. Additionally, the oxidation of other non-fluorinated organic materials, facilitated by radical interactions generated during cavitation, likely played a role. However, the high COD also indicated significant competition for reactive radicals, which could potentially reduce the efficiency of PFAS mineralization.

Studies have shown that natural dissolved organic matter (DOM), such as humic acid (HA) and fulvic acid (FA), has a limited effect on PFAS degradation due to their nonvolatile and weakly surface-active nature (Cheng et al. 2008). However, the presence of hydrocarbon-based contaminants and anionic surfactants can compete for adsorption at the bubble-water interface, further decreasing degradation efficiency, Fig. 6 (7–8) (Lin et al. 2015). AFFF, on the other hand, exhibited the highest COD levels among all matrices due to the concentrated surfactants and organic additives present. Despite this, post-treatment COD reductions were modest, reflecting the persistence of certain organic compounds and the inefficiency of sonolysis alone in fully mineralizing PFOS and other stable PFAS in AFFF. Furthermore, the foam layer formed during treatment likely acted as a physical barrier, limiting the access of radicals to the bulk solution and further restricting COD reduction. The reduced defluorination efficiency observed in AFFF-treated samples is consistent with findings that fluorocarbon tails of PFOS remain largely intact in the presence of organic species, as shown in previous studies on diluted AFFF solutions (Cheng et al. 2008). Additionally, while anionic surfactants such as sodium dodecyl sulfate (SDS) slow down PFOA sonolysis due to competitive adsorption, cationic surfactants can enhance PFOA degradation by increasing its concentration at the bubble-water interface and strengthening attractive forces (Lin et al. 2015).

The sonolysis process generates highly reactive •OH and H• during cavitation, which attacks PFAS molecules, breaking down carbon–fluorine (C–F) bonds and releasing fluoride ions. However, the presence of organic materials in the matrix competes for these reactive species, altering the degradation pathway. In SB, the high COD represents significant competition for these radicals, reducing the effectiveness of PFAS mineralization. This competition is less pronounced in GW due to its lower COD, allowing for more efficient radical interaction with PFAS molecules. For AFFF, the high COD, combined with the physical barrier created by foam formation, significantly impedes degradation, as evidenced by the modest COD reduction and limited fluoride release. These findings are consistent with prior research demonstrating that organic contaminants and surfactants influence PFAS degradation rates in aqueous environments (Cheng et al. 2008; Lin et al. 2015).

### Inorganic components

In GW, moderate levels of inorganic ions such as chloride and sulfate enhance degradation by stabilizing cavitation bubbles and promoting radical formation, leading to higher fluoride release (0.51 to 1.78 mg/L after 12 h) (Appendix Table 6) despite low PFAS concentrations. This aligns with studies showing that anions influence PFAS sonolysis efficiency based on their interaction with the bubble-water interface. For example, chloride (Cl^-^) improves sonolysis by increasing PFAS hydrophobicity and accumulation at the interface (Cheng et al. 2008). Additionally, sulfate (SO_4_2^-^) plays a dual role depending on its concentration, potentially enhancing or hindering degradation (Lin et al. 2015). In contrast, bicarbonate (HCO_3_^-^) has been reported to slow down PFAS sonolysis by altering interfacial pyrolysis conditions, though other studies suggest it may enhance degradation via CO_3_^-^• radicals, Fig. 6 (11) (Phan Thi et al. 2014; Zeidabadi et al. 2023).

SB samples benefit from high sulfate levels, which aid sulfonated PFAS degradation, but competition for radicals reduces overall efficiency. Extended treatment results in significant fluoride release (8.71 mg/L after 12 h) (Appendix Table 6), reflecting partial mineralization. Previous findings indicate that sulfate-assisted sonolysis can either enhance or inhibit PFAS degradation based on concentration-dependent effects (Lin et al. 2015). In AFFF, the dominance of PFOS and foam formation limits the effectiveness of inorganic ions, resulting in minimal fluoride release (0.64 to 1.37 mg/L after 12 h) (Appendix Table 6). The presence of perchlorate (ClO_4_^-^) has been shown to increase negative surface potential, reducing bubble agglomeration and increasing the available interfacial area for PFAS pyrolysis (Fig. 6 (9)) (Cheng et al. 2008). However, the foam layer in AFFF restricts cavitation efficiency and hinders radical interaction with PFAS. Organic components, however, primarily hinder degradation. High organic loads in SB and AFFF compete with PFAS for hydroxyl radicals, reducing mineralization efficiency. In SB, COD reductions indicate partial breakdown of organics, while in AFFF, surfactants form a foam layer that impedes cavitation and limits PFAS interaction with radicals. In contrast, GW’s low organic content facilitates more direct radical interaction with PFAS, supporting higher degradation efficiency.

## Energy utilization and PFAS degradation efficiency

The degradation of PFAS in complex matrices such as SB, GW, and AFFF presents unique challenges. These challenges stem from matrix-specific complexities, competing organic loads, and the inherent stability of PFAS molecules due to their strong C–F bonds. This section evaluates the energy distribution in ultrasonic reactors, quantifying its role in achieving PFAS degradation through defluorination and COD reduction. In addition, the specific energy consumption was normalized to the mass of fluoride removed in each matrix to better assess treatment efficiency and benchmark ultrasound performance against competing technologies.

### Total energy input

For all matrices, a power density of 118 W/L, a volume of 10 L, and a treatment time of 12 h is the total energy input. Therefore, the energy supplied to the ultrasonic reactor is derived from the following:

5 $${E}_{\mathrm{total}}=P \times V \times t$$

where *P* is power density (w/L), *V* is volume, and *t* is treatment time (s), hence the above resulted in a total energy input of 50,976 kJ.

### Energy utilization for defluorination

Defluorination energy, essential for breaking PFAS’s strong C–F bonds, is 544 kJ/mol (Vakili et al. 2024). Therefore, the energy of defluorination is calculated as follows:

6 $${E}_{\mathrm{def}}= \frac{\Delta \left[{F}^{-}\right]\times V}{{M}_{F}}\times {E}_{\mathrm{CF}}$$

Δ[*F*^−^] is the change in fluoride ion concentration released (mg/L), *M*_*F*_ is molar mass of fluoride (19 g/mol) and *E*_CF_ is the bond energy for C–F bonds (544 kJ/mol).

### Energy for COD reduction

COD reduction involves the oxidation of carbon bonds in organic matter, requiring energy to break these bonds. For simplicity, assume the dominant bonds oxidized in COD are C–H bonds (the most common in organic loads). Therefore, the energy required is expressed as follows:

7 $${E}_{C}= \frac{\Delta \left[C\right]\times V}{{M}_{C}}\times {E}_{C \mathrm{bond}}$$

Δ[*C*] is the change in carbon content (mg/L), *M*_*c*_ is molar mass of the representative organic compound (g/mol), and *E*_*C*bond_ is the bond energy of the specific C bond being oxidized (C–H bonds: ~ 412 kJ/mol).

The energy distribution in PFAS degradation (see Appendix Table 9) highlights the interplay between defluorination, organic matter oxidation (COD reduction), and energy dissipation. In the ultrasonic reactor, the total energy input is distributed among breaking strong C–F bonds (defluorination), oxidizing organic contaminants (COD reduction), and secondary effects like heat loss and cavitation. Among the matrices, SB utilized the high energy for COD reduction (13,204.67 kJ) due to its significant organic load (385 mg/L COD reduction). In contrast, GW exhibited minimal COD reduction (1 mg/L), consuming 342.97 kJ. AFFF, a complex matrix, showed moderate COD reduction (49 mg/L) and utilized 16,809.94 kJ for COD, leaving 8676.34 kJ unaccounted, indicating both physical (foam) and chemical (C–F bond) resistance to degradation. Defluorination is a key mechanism in PFAS degradation, directly linked to the breakdown of C–F bonds and fluoride release. SB released the highest fluoride concentration (5 mg/L) with *E*_def_ = 2.72 kJ, indicating better PFAS degradation. However, the high COD in SB competes for reactive radicals like ⋅OH, reducing the energy available for defluorination. AFFF, with a fluoride release of 0.6 mg/L and *E*_def_ = 1.72 kJ, demonstrates slow PFAS degradation due to foam interference and a chemically stable matrix. Groundwater, with 1.39 mg/L fluoride release, shows limited PFAS breakdown due to low organic content and simpler matrix composition. The competition between COD reduction and defluorination reveals that high organic loads in SB and AFFF matrices hinder PFAS degradation by consuming energy and reactive species, underscoring the importance of pre-treatment to reduce COD for efficient PFAS remediation.

The extremely low degradation rate for AFFF is due to the highest consumption of energy used for the degradation of COD plus the extensive foam formation due to the high surfactant content. Hence with the high concentration of surfactants in AFFF samples, there is a tendency to merge several nano/micro bubbles into macro bubbles instead of bubble implosion causing lower destruction rate. Authors are currently using molecular dynamic (MD) simulations to provide a theoretical explanation to merging of nano/micro bubbles instead of implosion. Although defoaming measures were not tested in this study, future work will explore mechanical skimming, antifoam additives, or pulsed ultrasound to reduce foam interference and improve cavitation efficiency in AFFF systems.

### Energy consumption per fluoride removed

To contextualize the energy efficiency of ultrasound treatment across different matrices, the energy input was normalized based on the mass of fluoride released. The specific energy consumption per gram of fluoride removed (kJ/g F^-^) was calculated using the following expression:

8 $${E}_{\mathrm{norm}}= \frac{{E}_{\mathrm{total}}}{\Delta \left[{F}^{-}\right]\times V/1000}$$

where ${E}_{\mathrm{norm}}$ is in kJ/g F^−^, ${E}_{\mathrm{total}}$ is the total energy input (kJ), Δ[F^−^] is the fluoride ion released (mg/L), and $V$ is the volume in liters. The calculated specific energy consumption values were: SB = 5852 kJ/g F^-^, GW = 28,625 kJ/g F^-^, and AFFF = 37,206 kJ/g F^-^. As presented in Appendix Table 9, SB was the most energy-efficient matrix, owing to its higher fluoride release and fewer interferences from foam or organic loads. In contrast, GW and AFFF exhibited significantly higher energy demands due to either low PFAS concentrations (GW) or foam-induced cavitation suppression (AFFF), both of which limited degradation efficiency.

To benchmark ultrasound performance, these values were compared to those reported for other PFAS destruction technologies. Recent plasma-based systems treating still-bottom matrices have shown energy demands of approximately 1885 kWh/m^3^ (≈ 6800 kJ/g F^-^, assuming 1 mg/L PFAS) (Higgins et al. 2025). Electro-oxidation technologies using advanced electrodes like Ti_4_O_7_ and BDD have reported energy consumption ranging from 3.6 to 19.9 kWh/m^3^ (≈ 13,000–72,000 kJ/g F^-^) (Ryan et al. 2021; Wang et al. 2021).

In comparison, our ultrasound-treated SB matrix required 5852 kJ/g F^-^, falling well within these benchmark ranges. Despite the indirect and radical-based mechanism of ultrasound, which is often less energy-efficient in dilute or foam-rich matrices, this result highlights its competitive energy efficiency for high-strength PFAS waste. Furthermore, ultrasound systems offer operational advantages such as chemical-free treatment, modularity, and integration potential with other advanced oxidation processes, supporting their use in decentralized or site-specific remediation applications.

## Summary and conclusion

This study investigated the efficacy of ultrasound technology for degrading PFAS in three environmental matrices: SB, GW, and AFFF. The degradation efficiency (DE) varied across these matrices, influenced by initial concentrations, molecular structures, and matrix complexity. SB samples, with initial PFAS concentrations of 12–18 mg/L, exhibited significant defluorination, reaching fluoride concentrations of 8.71 mg/L after 12 h. This aligns with high DE values (up to 65%) observed via LC–MS, NMR, and TOF-AOF. GW samples, with lower initial PFAS concentrations (~ 0.5 mg/L), showed moderate fluoride release (0.54 to 1.78 mg/L) but high DE, demonstrating the effectiveness of ultrasound in low-concentration matrices. Conversely, AFFF samples, dominated by PFOS at 6800 mg/L, showed minimal degradation and fluoride release (0.75 to 1.37 mg/L). Foam formation likely reduced cavitation efficiency, preventing substantial PFOS breakdown. These results indicate that ultrasound is more effective in lower-concentration or non-foaming PFAS matrices.

The reduction in soluble metal ion concentrations post-sonolysis suggests complexation or removal mechanisms. PFAS and their degradation products likely formed stable metal complexes, reducing detectable metal concentrations. The decline in iron and manganese levels in SB and GW samples may indicate redox interactions or complexation with PFAS degradation intermediates. Calcium reductions suggest possible precipitation (e.g., CaF_2_ formation), explaining decreased solubility post-treatment. Fluoride release confirmed partial defluorination, particularly in SB samples, reinforcing the effectiveness of ultrasound in mineralizing PFAS. Unexpected sulfate reduction contradicts expected sulfonic acid degradation pathways, necessitating further investigation into potential precipitation or adsorption mechanisms. Similarly, chloride reduction lacks a clear transformation pathway under ultrasonics, suggesting adsorption or reactor interactions rather than chemical degradation.

Overall, ultrasound treatment demonstrated effective PFAS degradation in SB and GW matrices but was limited for AFFF due to PFOS resistance and foam formation. Metal and anion interactions suggest complex transformations, but additional studies are needed to clarify mechanisms. Enhancing treatment conditions and integrating complementary methods could improve the applicability of ultrasound for PFAS remediation in diverse environmental matrices.

The scalability of ultrasound-based PFAS degradation remains a key consideration for future applications. While bench-scale experiments have shown promising removal efficiency, transitioning to pilot or full-scale systems will require optimization of reactor design, ultrasound transducer placement, and energy delivery strategies.

Although ultrasound technologies are increasingly being explored for environmental applications, their economic viability at larger scales depends on improving energy efficiency, minimizing operational costs, and integrating them into existing treatment trains. Future research should focus on developing techno-economic assessments and life cycle analyses to evaluate the full feasibility of deploying ultrasound systems for PFAS treatment in real-world settings.

## Data Availability

All the data used are included in the manuscript.

## Appendix

See Table 2

**Table 2:** Raw ground water sample information

Compounds	GW [mg/L]	Ratio (%)	Cumulative percentage (%)
6 2 FTS	0.361	42.3	42.3
PFPeA	0.116	13.6	55.9
PFHxS	0.114	13.3	69.2
PFHxA	0.097	11.4	80.6
PFOA	0.074	8.7	89.3
PFHpA	0.048	5.7	94.9
PFBA	0.019	2.2	97.1
PFBS	0.013	1.5	98.6
PFPeS	0.012	1.4	100
Total PFAS	0.853		

See Table 3

**Table 3:** Raw still bottom sample information

Compound name	Concentration (mg/L)	Ratio (%)	Cumulative percentage (%)
PFOA	405	68.31	68.31
PFHxS	69.3	11.69	80.00
PFHxA	29.2	4.93	84.93
PFPeS	20.1	3.39	88.32
PFHpA	18.7	3.15	91.47
PFOS	15	2.53	94.00
PFBS	9.08	1.53	95.53
PFPeA	7.93	1.34	96.87
6:2FTS	4.8	0.81	97.68
8:2FTS	4.15	0.70	98.38
PFNA	3.02	0.51	98.89
PFDA	2.13	0.36	99.25
PFHpS	1.82	0.31	99.56
PFBA	0.967	0.16	99.72
Others PFAS	1.67081	0.28	100.00
Total PFAS =	592.9		

See Table 4

**Table 4:** Raw AFF sample information

Compounds	AFFF [mg/L]	Ratio (%)	Cumulative percentage (%)
PFOS	6802.165	0.78	77.84
PFHxS	1025.645	0.12	89.58
PFBS	189.98	0.02	89.6
PFPeS	139.535	0.02	89.62
HFPO-DA	87.995	0.01	89.63
PFHxA	86.595	0.01	89.64
PFBA	60.775	0.01	89.64
PFHpS	59.415	0.01	89.65
PFDoA	56.415	0.01	89.66
PFOA	54.905	0.01	89.67
PFHpA	50.03	0.01	89.67
PFDA	45.008	0.01	89.68
PFNA	40.05	0.01	89.68
4 2 FTS	22.64	0.01	89.68
PFPeA	13.87	0.01	89.68
8 2 FTS	4.48	0.01	89.68
9Cl-PF3ONS	3.99	0.01	89.68
11Cl-PF3OUDS	1.23	0	89.68
Total PFAS	8738.52		

See Table 5

**Table 5:** Pseudo-first-order rate constants, half-lives, and coefficients of determination (*R*^2^) for PFAS degradation across different water matrices (6-h treatment)

Matrix	PFAS compound	Rate constant (k, h^-1^)	Half-life (*t*_1_/_2_, h)	*R* ^2^
GW	6:2 FTS	0.1682	4.12	0.7578
GW	PFBA	0.1698	4.08	0.7986
GW	PFBS	0.3727	1.86	0.867
GW	PFHpA	0.3186	2.18	0.9975
GW	PFHxS	0.5518	1.26	0.9099
GW	PFOA	0.4289	1.62	0.9168
GW	PFPeA	0.4524	1.53	0.9773
GW	PFPeS	0.411	1.69	0.9221
AFFF	PFBS	0.0187	37.06	0.9206
AFFF	PFHpS	0.0231	30	0.6286
AFFF	PFHxA	0.0013	533.08	0.0156
AFFF	PFHxS	0.009	77	0.6478
AFFF	PFOS	0.013	53.31	0.762
AFFF	PFPeS	0.0192	36.09	0.8552
SB	PFBS	− 0.0896	7.73	0.6962
SB	PFHpA	0.0628	11.03	0.8798
SB	PFHxA	0.1358	5.1	0.941
SB	PFHxS	0.0457	15.16	0.9806
SB	PFOA	0.0959	7.22	0.9164
SB	PFOS	0.1254	5.53	0.8545
SB	PFPeS	0.0738	9.39	0.994

See Table 6

**Table 6:** Change in fluoride, sulfate, and chloride concentrations after sonolytic treatment for three PFAS matrices

Sample ID	Initial concentration (mg/L)			Final concentration (mg/L)		
	Fluoride	Sulfate	Chloride	Fluoride	Sulfate	Chloride
6-h GW	0.54	0.00	3.63	1.39	0.00	3.67
12-h GW	0.51	0.00	3.51	1.78	0.00	3.57
6-h SB	0.00	842.53	6958.35	5.00	363.96	4026.44
8-h SB	0.00	449.97	5368.88	6.02	371.01	5231.69
9-h SB	0.00	298.31	3012.27	5.77	247.91	2249.82
12-h SB	0.00	347.39	2585.27	8.71	255.74	2006.86
6-h AFFF	0.75	0.00	0.00	1.03	0.00	0.00
12-h AFFF	0.64	0.00	0.00	1.37	0.00	0.00

See Table 7

**Table 7:** Changes in NMR CF3 concentration and TOF-AOF fluorine levels across different treatment durations

Experiment list	NMR (CF_3_ mM)	TOF (AOF fluorine mg/L)
6-h GW before	0.000198	1.70
6-h GW after	0.000084	0.65
12-h GW before	0.000236	1.70
12-h GW after	0.000026	0.21
6-h SB before	1.966996	34.00
6-h SB after	1.142370	13.00
8-h SB before	2.127902	27.00
8-h SB after	1.614355	16.00
9-h SB before	1.170259	13.00
9-h SB after	0.703476	6.60
12-h SB before	1.567534	17.00
12-h SB after	0.561896	7.90
6-h AFFF before	0.003756	41.00
6-h AFFF after	0.003545	39.00
12-h AFFF before	0.004244	47.00
12-h AFFF after	0.003918	40.00

See Table 8

**Table 8:** Comparison of defluorination and fluoride yield in GW, SB, and AFFF samples

Sample	Fluoride_released (mg/L)	Defluorination (mg/L)	Unaccounted fluorine (mg/L)	Defluorination (%)	Fluoride yield (%)
6-h GW	0.54	1.05	0.51	61.76	31.76
12-h GW	1.39	1.49	0.1	87.65	81.76
6-h SB	8.71	21	12.29	61.76	25.62
8-h SB	6	11	5	40.74	22.22
9-h SB	6.4	6.4	0	49.23	49.23
12-h SB	8.1	9.1	1	53.53	47.65
6-h AFFF	1.03	5.4	4.37	13.17	2.51

See Table 9

**Table 9:** Comprehensive energy analysis for PFAS treatment

Matrix	Fluoride released (mg/L)	Energy for defluorination (kJ)	COD reduction (mg/L)	Energy for COD reduction (kJ)	Energy per gram of F^-^ removed (kJ/g)
Groundwater (GW)	1.39	0.76	1.00	342.97	28,625
Still bottom (SB)	5.00	2.72	385.00	13,204.67	5,852
Aqueous film forming foam (AFFF)	1.39	0.76	1.00	342.97	37,206
